# A multiple detection method for distinguishing gene mutations based on melting curves of extended quenching probes

**DOI:** 10.1016/j.heliyon.2022.e11856

**Published:** 2022-11-26

**Authors:** Wang Jianping, Liu Zipeng, Pan Tengfei, Zhang Song

**Affiliations:** a R&D Department, Guangzhou Biotron Biotechnology Co., Ltd., Guangzhou 510700, Guangdong, China; b Guangzhou Institute of Pediatrics, Guangzhou Women and Children's Medical Center, Guangzhou Medical University, Guangzhou 510623, Guangdong, China

**Keywords:** EGFR gene, Gene mutations, Melting curve, Multiple DNA target Detection, Non-small cell lung cancer

## Abstract

Conventional PCR methods can detect only a few targets simultaneously and do not fulfill most clinical requirements, especially those for detecting plasma circulating DNA. By designing characteristic universal fluorescent probes, combining multiplex PCR with the invasive reaction, and analyzing the resulting differences in the melting curves formed by extension with double-stranded probes, we developed a new method to distinguish between three mutations in the same fluorescent channel and nine mutations in three fluorescent channels in a single tube. After optimization, this method was used to distinguish between 27 mutations using only three reactions, and mutations representing as low as 0.2%–0.5% of DNA could be detected, even when up to nine mutations were present at the same time. Testing of nine clinical samples, including three L858R-positive, four 19 del-positive, and two L861Q-positive samples, showed consistent results with digital PCR tests. Compared with the conventional PCR method, our method expands the capabilities of fluorescence detection by achieving multiplex detection in a single-tube, thereby providing a simple, low-cost tool for clinical applications.

The detection of mutations in specific genes from DNA extracted from tumors can effectively improve treatment for patients by allowing for personalization of their therapy (Reck et al., 2020; Zhang et al., 2020). In reality, tumor samples are mainly derived from tumor tissue and blood (Nie et al., 2015; Ni et al., 2017). However, the acquisition of tumor tissues is subject to strict regulations, and *ex vivo* tissues do not accurately reflect tumor development; thus, the benefits for patients are limited. Tumor tissue-derived nucleic acids can be detected in blood, as apoptotic cells from tumor tissue release genetic material into the blood (Fleischhacker and Schmidt, 2008). Nevertheless, the total amount of nucleic acid and the proportion of mutated nucleic acids in plasma vary greatly (Diehl et al., 2008). It has been reported that the total amount of nucleic acid in 5 ml plasma varies from several to several hundred micrograms, and the proportion of mutated nucleic acid ranges from 0.1% to 50% (Diehl et al., 2008; Fleischhacker and Schmidt, 2008). This poses a challenge for mutation detection in tumor tissue-derived nucleic acids, and there is a great need for more sensitive methods.

With the progression of molecular diagnostic technologies, a variety of methods have been developed for gene mutation detection in the clinic (Martin et al., 2022; Tian et al., 2022). For example, high resolution melting technology can be used to identify the characteristic melting curve generated by a template containing a mutation. The detection limit of this method is 3%–5%, which does not meet the detection requirements for clinical samples, especially for blood samples (Ginart et al., 2019; Nikodem et al., 2021). The amplification refractory mutation system (ARMS) PCR can detect gene mutations with frequencies of 1%, which is acceptable for most clinical samples (Zhang et al., 2019; Okada et al., 2022). Digital PCR has a high limit of detection, but can only be used to detect one or a few mutation sites and is unsuitable for clinical detection of multiple mutation sites (Tanzima Nuhat et al., 2018). The “Tube-Lab”, which is based on a gold nanoparticle indicator, can detect mutations with frequencies as low as 0.1% and has been validated in plasma samples but has limited applications due to its requirement for a separate tube for each mutation site (J. Wang et al., 2017).

Clinical research has identified an increasing number of mutation targets with various levels of clinical significance for cancer diagnosis and treatment. The epidermal growth factor receptor (EGFR) gene plays an important role in the development of non-small cell lung cancer (NSCLC) (Malapelle et al., 2016; L. Wang et al., 2017). When tumor tissue from NSCLC patients harbors activating EGFR gene mutations, these can be treated with tyrosine kinase inhibitors (TKIs), such as gefitinib and afatinib (Beagan et al., 2020). The L858R mutation in the EGFR gene is a good biomarker for drug targeting (Heng et al., 2019; Pratama et al., 2019). In contrast, the T790M mutation does not respond to drug targeting, but together with the C797S mutation, it shows cis-resistance and trans-sensitivity to third-generation targeting drugs (Madic et al., 2018; Arulananda et al., 2019). Therefore, before EGFR-TKIs are taken by patients with NSCLC, it is necessary to determine whether the tumor tissue harbors gene mutations and identify the type of mutation in the EGFR gene (Joy et al., 2020). Thus, the growing number of roles discovered for different mutations increases the clinical value of discriminating between types of mutations.

When simultaneously discriminating between mutations, the traditional PCR method requires multiple tubes for parallel detection (Yanagita et al., 2016). This limitation is related to fluorescence channels and increases complexity and experimental costs (Zhang et al., 2021). Next-generation sequencing can handle a large amount of data and can be used to detect thousands of genes at one time but also has the disadvantages of being complex, expensive, and time consuming (Toledo et al., 2018; Yokouchi et al., 2020). Therefore, it is necessary to develop a low-cost and easy-to-use method to detect multiple mutation sites simultaneously. In this paper, we introduce a method that combines multiplex PCR and the invasive reaction. The invasive reaction is highly specific because the signal corresponding to the recognized sequence is produced only when the “invasive structure” is formed by the upstream and downstream probes with the target template (Liu et al., 2018; Xiang et al., 2018). The invasive reaction is widely used for detection of various nucleic acids, in particular gene mutations (Usami et al., 2008; Yamamoto et al., 2009). Our method uses the different melting peaks formed by the fluorescent signal molecules produced during the invasive reaction, which extend along the quenching probes (QPs) to further expand the fluorescence detection capacity, i.e., to detect three mutation sites in a single fluorescence channel. Using FAM, ROX, and CY5 fluorescence channels, nine mutation sites could be detected in the EGFR gene, not only providing more accurate results but also tackling the issues of sample scarcity and cost.

## 1 Materials and methods

### 1.1 Equipment and reagents

SLAN-96 Fluorescence PCR (Shanghai Hongshi Medical Technology Co., Ltd., catalogue number: SLAN-96S, Shanghai, China), the Naica™ Crystal digital PCR system (Stilla Technologies Inc., France), a Nanodrop ultraviolet spectrophotometer (Thermo Fisher Scientific, catalogue number: ND-ONE-W, USA), and a two-person biosafety cabinet and chemical hood (Shandong Biobase Co., China) were used.

Taq DNA (Shanghai Promega Co., Ltd., catalogue number: M1661S, Shanghai, China) and a Nucleic acid extraction kit and Afu endonuclease (Guangzhou Biotron Technology Co., Ltd., China) were used. All other chemicals and solvents (analytical reagent grade) were purchased from Sigma (St. Louis, MO, USA). The target sequences of the EGFR gene were retrieved from the NCBI (https://www.ncbi.nlm.nih.gov/) and COMSIC databases (https://cancer.sanger.ac.uk/cosmic). Twenty-seven mutations were located on exons 18–21 (Table 1). Primers and probes compatible with different gene sequences were designed using IDT DNA online software (https://sg.idtdna.com/pages/tools/) and Oligo 7 design software (http://www.oligo.net/), and the sequences are shown in Supplementary Table S1. Primers and probes were obtained by Sangon Biotech, Co., Ltd., (Shanghai, China) and plasmid was obtained from Bioligo Co., Ltd., (Shanghai, China).

**Table 1:** The detecting information of EGFR mutation sites.

Tube Number	Fluorescent Channel	Tm/°C	Mutation	Nucleotide Variation	Extron	Cosmic ID
Tube 1	FAM-Tm1	69.5	G719A	2156G>C	18	6239
	FAM-Tm2	74.5	G719S	2155G>A	18	6252
	FAM-Tm3	85.0	G719C	2155G>T	18	6253
	ROX-Tm1	71.2	E746_A750del (1)	2235_2249del15	19	6223
	ROX-Tm2	78.5	E746_A750del (2)	2236_2250del15	19	6225
	ROX-Tm3	87.5	L747_P753 > S	2240_2257del18	19	12370
	CY5–Tm1	69.5	E746_T751 > I	2235_2252 > AAT (complex)	19	13551
	CY5–Tm2	76.5	E746_T751del	2236_2253del18	19	12728
	CY5–Tm2	86.5	E746_T751 > A	2237_2251del15	19	12678
Tube 2	FAM-Tm1	69.5	E746_S752 > A	2237_2254del18	19	12367
	FAM-Tm2	74.5	E746_ S752 > V	2237_2255 > T (complex)	19	12384
	FAM-Tm3	85.0	E746_ S752 > D	2238_2255del18	19	6220
	ROX-Tm1	71.2	L747_A750 > P	2238_2248 > GC (complex)	19	12422
	ROX-Tm2	78.5	L747_T751 > Q	2238_2252 > GCA (complex)	19	12419
	ROX-Tm3	87.5	L747_E749del	2239_2247delTTAAGAGAA	19	6218
	CY5–Tm1	69.5	L747_T751del	2239_2253del15	19	6254
	CY5–Tm2	76.5	L747_S752del	2239_2256del18	19	6255
	CY5–Tm2	86.5	L747_A750 > P	2239_2248TTAAGAGAAG>C (complex)	19	12382
Tube 3	FAM-Tm1	69.5	T790M	2369C>T	20	6240
	FAM-Tm2	74.5	L747_P753 > Q	2239_2258 > CA (complex)	19	12387
	FAM-Tm3	85.0	S768I	2303_G>T	20	6241
	ROX-Tm1	71.2	H773_V774insH	2319_2320insCAC	20	12377
	ROX-Tm2	78.5	D770_N771insG	2310_2311insGGT	20	12378
	ROX-Tm3	87.5	L858R	2573T>G	21	6224
	CY5–Tm1	69.5	C797S	2389T>A	20	6493937
	CY5–Tm2	76.5	V769_D770insASV	2307_2308insGCCAGCGTG	20	12376
	CY5–Tm2	86.5	L861Q	2582T>A	21	6213

### 1.2 DNA extraction from clinical samples

DNA was obtained from clinical residual plasma samples using the nucleic acid extraction kit. Nucleic acid concentration and purity were detected using the UV spectrophotometer. 1 × TE buffer (10 mmol/L Tris-HCl, 1 mmol/L EDTA (pH 8.0)) was used to dilute samples to 10–30 ng according to the concentration obtained from the UV spectrophotometer.

### 1.3 Detection of multiple mutation sites

A 40-μL reaction system was prepared that included the following: 1 × PCR buffer, 50–200 nmol/L primers (P-Fs, P-Rs), 100–500 nmol/L upstream probe (UP), 200–600 nmol/L fluorescent probe (DP), 500 nmol/L QP, 2.5 U Taq DNA polymerase and 80 ng Afu endonuclease. The working concentrations of all primers and probes are listed in detail in Supplementary Table S2. The 1 × PCR buffer contained 15 mmol/L Tris-HCl (pH 8.5), 30 mmol/L NaCl, 6 mmol/L MgCl_2_, 0.05% Tween-20, and 0.05% IGEPAL® CA-630. The reaction procedure was as follows: 45 cycles of 2 min at 95 °C, 10 s at 95 °C, 40 s at 69 °C; 15 min at 60 °C; 15 s at 42 °C; 15 s at 45 °C; and melting curve analysis at 60–95 °C. Different amounts of each sample were mixed with wild-type DNA and different proportions of corresponding plasmids. Clinical samples were analyzed by assessing characteristic melting curves and results were further validated using digital PCR.

## 2 Results and discussion

### 2.1 Detection principle

**Figure 1:**
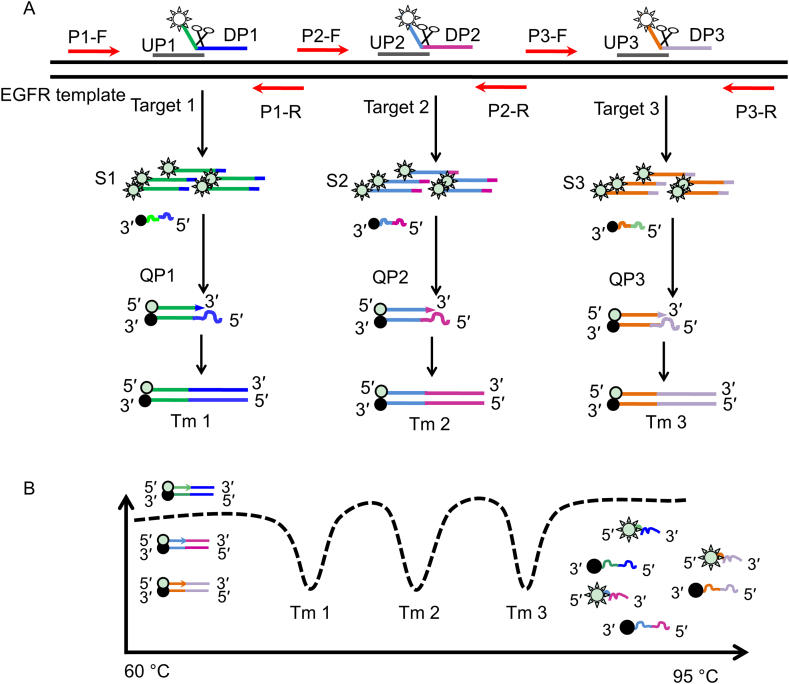
Schematic of the assay procedure for detecting EGFR mutations. 1A shows a schematic of the process for distinguishing between EGFR mutations. 1B shows the dynamic fluorescence intensity from 60 °C to 95 °C. Three melting peaks can be observed on a single fluorescence channel.

In order to detect different mutations in a single tube, multiplex PCR was combined with the nucleic acid invasion reaction, and the characteristic melting curves of the multiple fluorescent probes were used to identify three mutation sites in the corresponding fluorescence channels. The detection principle of this method is shown in Figure 1. EGFR mutation sites were used as an example (Figure 1A). Primers (P–F, P-R) were designed to amplify different targets. UPs and DPs were designed, with the UP sequence at the 3′ terminal end of the mismatched template and invading the first position of the complementary sequence between the DP and the template. The 5′ end of the DP sequence was unrelated to the target nucleic acid and was modified with a fluorescent group. The middle sequence of the DP was modified with a quenching group. The fluorescence of the DP was quenched when the probe was intact, but fluorescence was produced when the probe was cleaved. The target nucleic acid hybridized with the UP and DP to form a unique “invasive structure”, which was recognized and cleaved by Afu endonuclease to generate a signal molecule (S) with a fluorescent group that gradually accumulated as reaction time increased. The 3′ end of the QP was completely complementary to S, and each QP had a corresponding S. At low temperatures in the presence of DNA polymerase, the S hybridized with the QP and extended into a double-stranded structure with a specific melting peak (Tm). Different QPs have different Tm values, and when a target is present, its corresponding Tm value can be obtained by melting temperature analysis for the resulting S (Figure 1B) and used to determine whether there is a corresponding mutation.

### 2.2 Optimization of DPs and QPs

**Figure 2:**
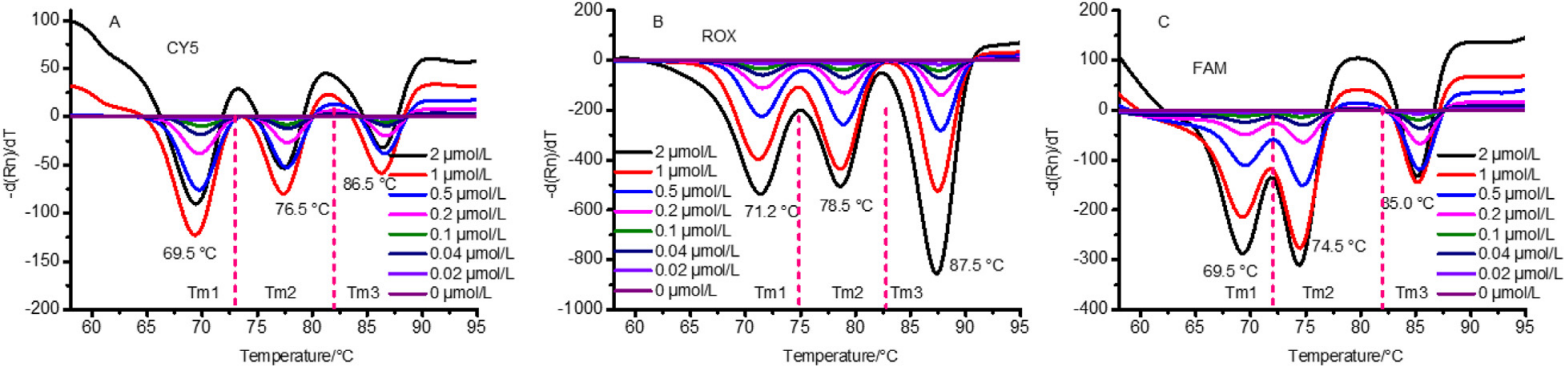
Results of different amounts of QPs and DPs in the FAM, ROX, and CY5 channels. 2A–2C represent the FAM, ROX, and CY5 channels, respectively. The three peaks correspond to Tm1, Tm2, and Tm3 for each fluorescence channel.

Because DPs and QPs have a significant impact on Tm, we optimized the amount of DPs and QPs in each of the three fluorescence channels. The final concentrations of QP in the experiment were 0, 0.02, 0.04, 0.1, 0.2, 0.5, 1, and 2 μmol/L, and equal concentrations of the corresponding DP were used (Figure 2). The results of Figures 2A-2C showed that as the amount of QP increased, Tm became more significant. However, the melting peak of each fluorescence channel was significantly different when the amount of QP exceeded 0.5 μmol/L, the case in point are the melting peaks of the ROX and FAM fluorescence channel, which are indicated in Figures 2B and 2C, respectively. Consequently, 0.5 μmol/L of QP and a corresponding amount of DP were used in the reaction. The Tm values for each channel are shown in Table 1. Tm1, Tm2, and Tm3 were 69.5, 76.5, and 86.5 °C in the CY5 fluorescence channel (Figure 2A), 71.2, 78.5, and 87.5 °C in the ROX fluorescence channel (Figure 2B), and 69.5, 74.5, and 85.0 °C in the FAM fluorescence channel (Figure 2C), respectively.

### 2.3 Multiple detection performance

**Figure 3:**
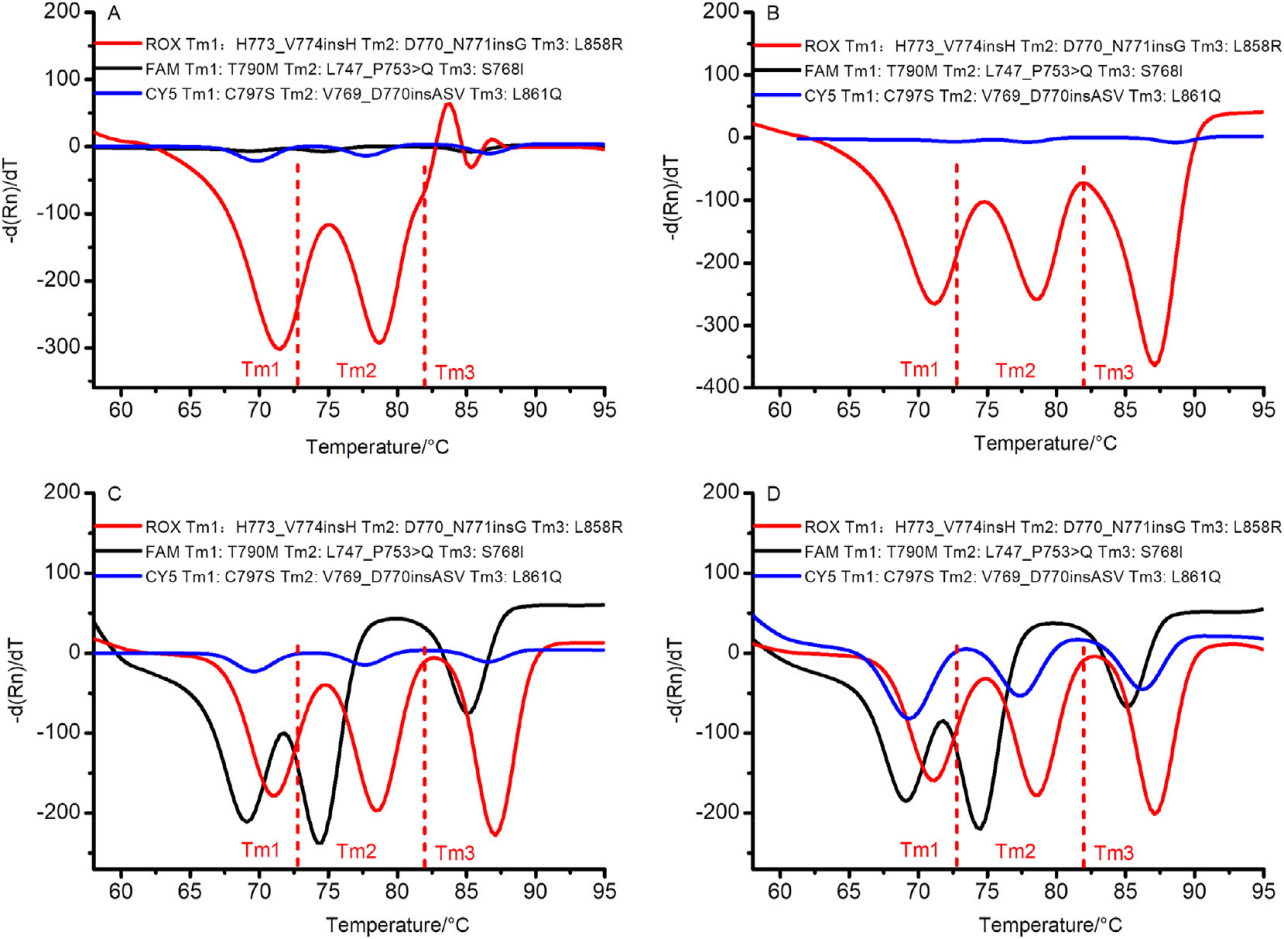
Simultaneous detection of different numbers of EGFR mutations. 3A shows the detection of two EGFR mutants. Figure 3B shows the detection of three EGFR mutants. Figure 3C shows the detection of six EGFR mutants. Figure 3D shows the detection of nine EGFR mutants. The three melting peaks (Tm1, Tm2, and Tm3) in the ROX, FAM, and CY5 channels correspond to H773_V774insH, D770_N771insG, L858R, T790M, L747_P753 > Q, S768I, C797S, V769_D770insASV, and L861Q, respectively.

Clinical samples may contain multiple mutations. Therefore, we tested the multiple detection capability of this method. Using tube 3 as an example, different numbers of mutation targets (2-plex, 3-plex, 6-plex, and 9-plex targets) were detected, and the results are shown in Figures 3A–3D. The results confirm that the method we established can simultaneously distinguish between mutation targets, whether the number of targets is two, three, six, or nine. Even up to a 9-plex reaction, each mutation could still be detected without errors, indicating that the method can be used to simultaneously detect multiple mutations.

### 2.4 Limit of detection (LOD)

**Figure 4:**
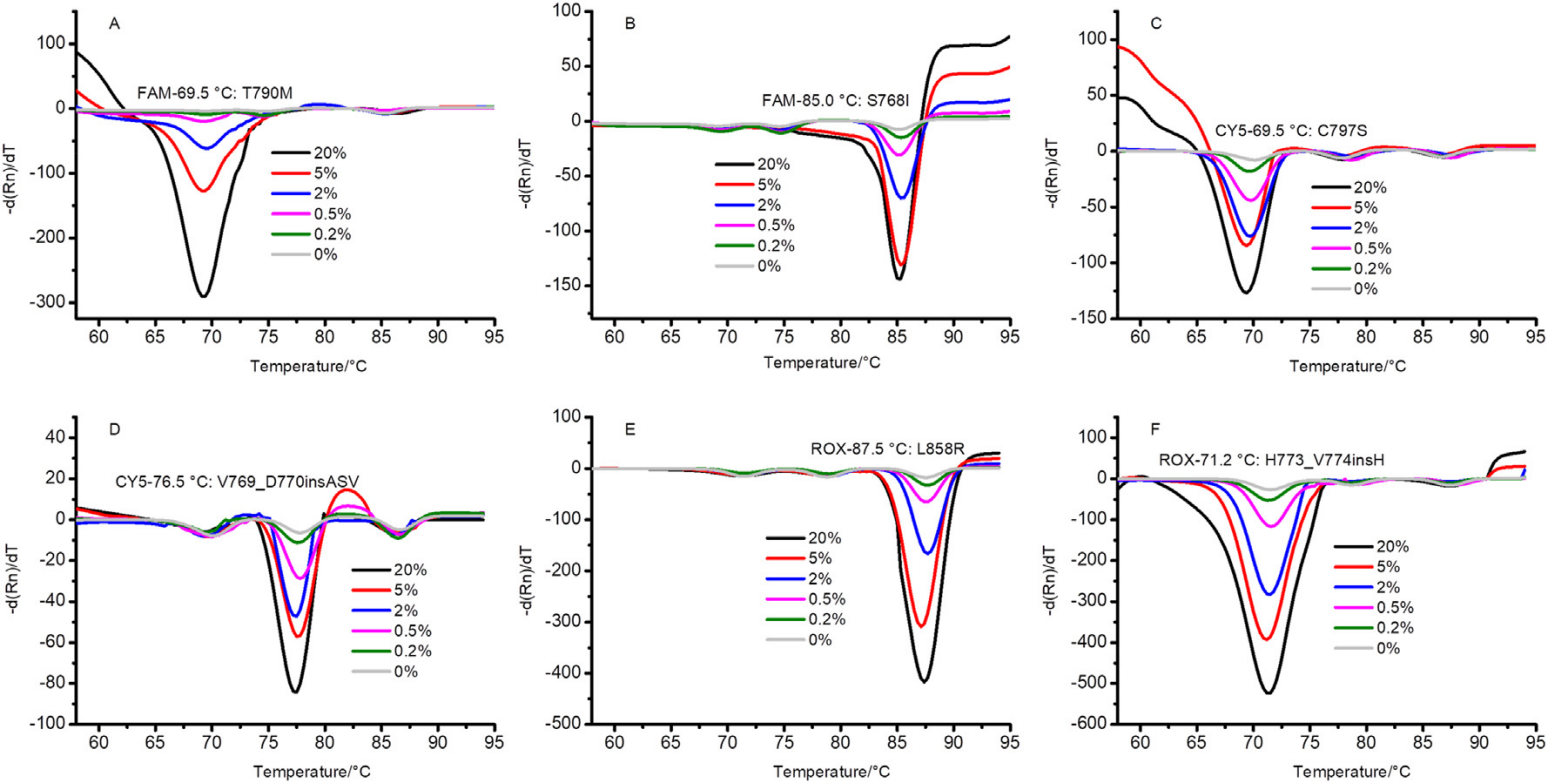
The LOD of the method for single-plex EGFR mutation. 4A–4F represent T790M (FAM), S768I (FAM), C797S (CY5), V769_D770insASV (CY5), H773_V774insH (ROX), and L858R (ROX), respectively. Samples with concentrations of 20%, 5%, 2%, 0.5%, 0.2%, and 0% were detected using this method.

To evaluate the LOD for the mutations, we detected single or multiple mutations using gradient dilution in tube 3. Two mutations were selected for gradient dilution in each fluorescence channel. Samples with concentrations of 20%, 5%, 2%, 0.5%, 0.2%, and 0% were used, and the results are shown in Figure 4. The results in Figures 4A–4F show that the minimum prevalence of combined mutations that could be detected by this method was >0.5%, but some mutations with lower prevalences could be detected, such as L858R, C797S, and H773_V774insH, for which 0.2% of the samples were distinguishable from the wild-type sample (0%). Therefore, the LOD for this method is >0.5%, which is better than most methods for detecting mutations based on qPCR (Chulakasian et al., 2010).

**Figure 5:**
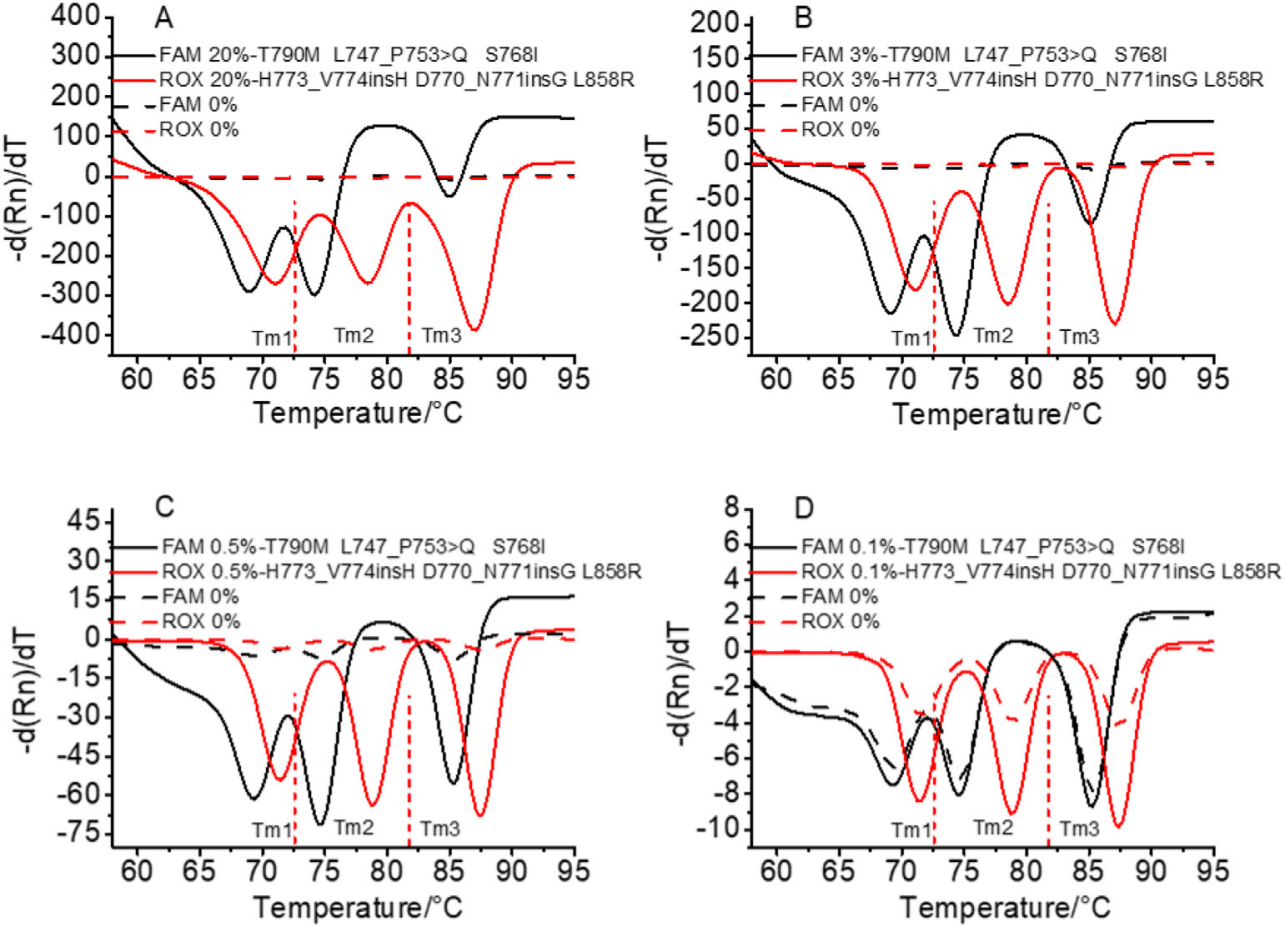
The LOD of the method for multiplexed EGFR mutations. 5A–5D show the detection of samples with concentrations of 20%, 3%, 0.5%, and 0.1%, which are marked with a solid line, and 0%, which is marked with a dotted line.

Next, six mutations from tube 3 (T790M, L747_P753 > Q, S768I, H773_V774insH, D770_N771insG, and L858R) in the FAM and ROX channels were selected, and the positive template was diluted to concentrations of 20%, 3%, 0.5%, 0.1%, and 0%. The results obtained using this method are shown in Figure 5. Figures 5A, 5B, and 5C show that positive results were detected at concentrations of 20%, 3%, and 0.5% (solid lines in Figures 5A–5C) for the sample containing six mutation sites when compared with wild-type samples (0%; dotted lines in Figures 5A–5C). Figure 5D shows that negative results were detected at 0.1% (solid line in Figure 5D) for the sample containing six mutation sites compared with wild-type samples (0%; dotted line in Figure 5D). These results reveal that this method can be used to detect samples with concentrations as low as 0.5% when six mutations are present at the same time. Thus, this method has a low LOD and suitable performance for multiple mutation detection. Existing gene mutation detection methods based on qPCR rarely achieve an LOD as low as 0.2%, and hardly any existing method based on qPCR can distinguish between multiple mutations (Ho et al., 2014; Feng et al., 2017).

To verify the precision of this method, samples with concentrations of 0.2% (L858R), 0.5% (D770_N771insG) and 0% were chosen for each of two mutation sites from tube 3, and eight replicates were performed for each sample. As shown in Supplementary Figure S1, the mean intensities ± standard deviations of the melting peaks were −33.3 ± 2.64 for 0.2% L858R and −32.4 ± 1.49 for 0.5% D770_N771insG and were significantly different from those of the negative sample (−16.2 ± 1.32 and −18.2 ± 1.52). These results showed that our method has high precision for sample detection.

### 2.5 Analysis of clinical samples

**Figure 6:**
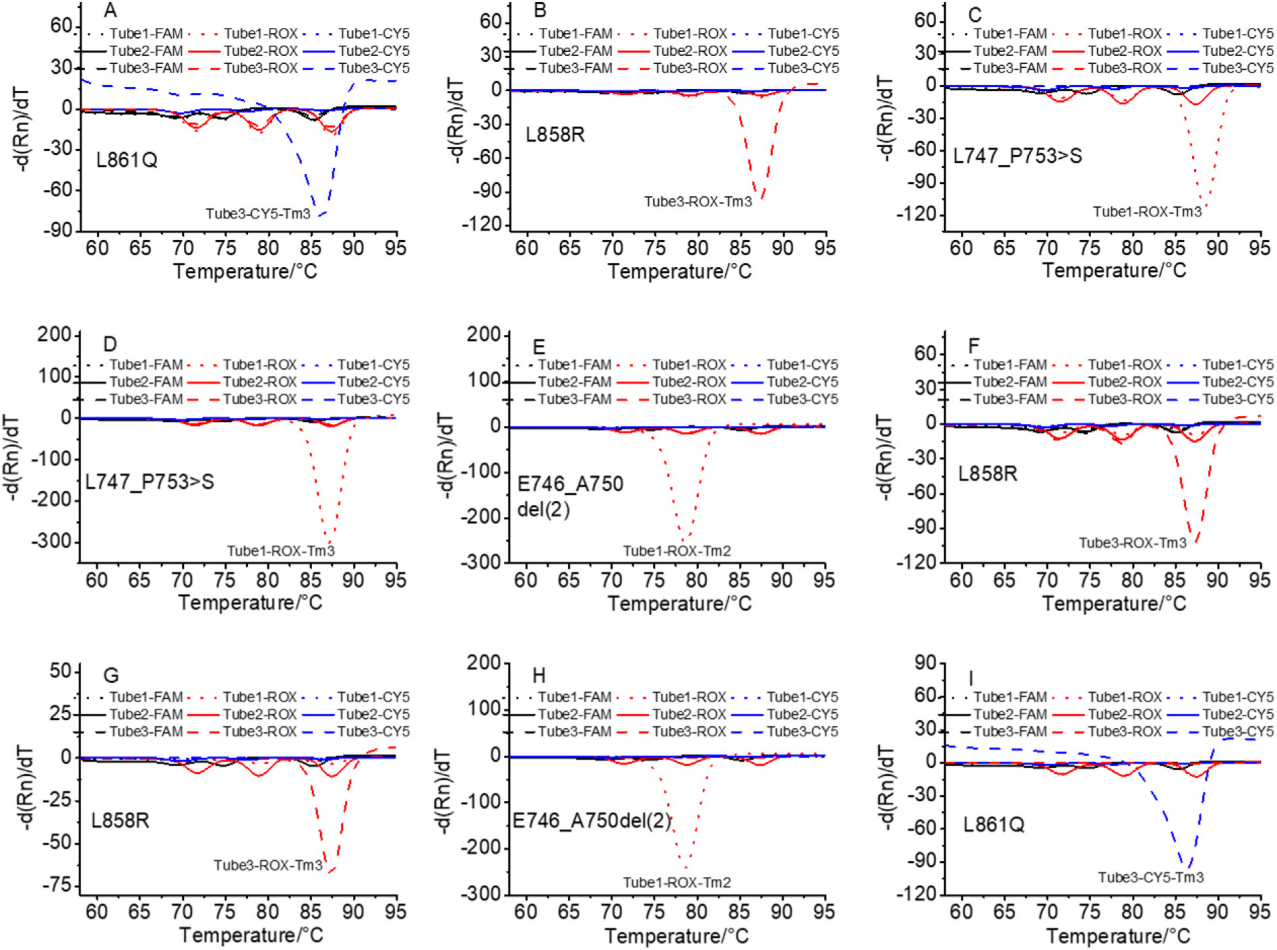
Results of nine clinical samples detected using this method. 6A–6I represent the results of samples 1–9. Of the nine clinical samples, three cases were L858R-positive, four cases were 19 del-positive (two cases contained L747_P753 > S and two cases contained E746_A750del (2)), and two cases were L861Q-positive.

**Figure 7:**
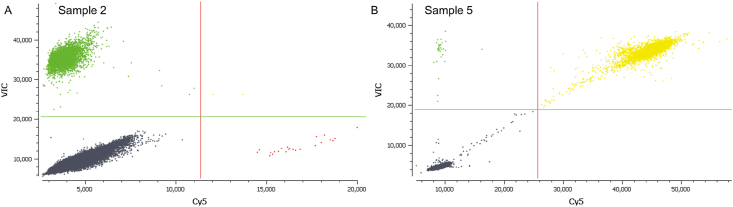
Results of digital PCR detection of samples 2 and 5. 7A: Sample 2 was L858R-positive. 7B: Sample 5 was E746_A750del (2)-positive.

Nine clinical samples from NSCLC patients were analyzed to verify the feasibility of this method for biological sample detection, as shown in Figure 6. Figures 6A–6I show the detection results of samples 1–9 (Supplementary Table S3), of which three were L858R-positive (2573T>G), four were 19 del-positive (two samples were L747_P753>S-positive (2240_2257del18) and two samples were E746_A750del (2)-positive (2236_2250del15)), and two were L861Q-positive (2582T>A). The results for these nine samples detected using our method were consistent with those of ARMS-PCR (Supplementary Figure S2), which is widely used for clinical diagnosis (Zhang et al., 2019; Okada et al., 2022). To further confirm the reliability of our results, samples 2 and 5 were verified by digital PCR, and the results were showed in Figures 7A and 7B, respectively. The results of samples 2 and 5 were consistent with those of our method (Sample 2: L858R-positive, Sample 5: E746_A750del (2)-positive), indicating that this method can be used for clinical mutation detection.

## 3 Conclusion

Molecular diagnosis-assisted targeted therapy for NSCLC is among the most successful applications of cancer therapies (Yung et al., 2009; Hu et al., 2012). As personalized targeted drugs and the therapeutic effects of different mutation types are developed and understood, clinical genotyping will become more and more important (Watanabe et al., 2015; Akher et al., 2020). However, existing methods cannot fully meet the clinical needs (Diehl et al., 2008). In this article, we developed a new method based on multiple fluorescence and characteristic melting peaks that performs well in the detection of multiple gene mutations. The method can be used to identify 27 known mutation sites in the EGFR gene using only three tubes, which is a great improvement over existing methods. Our results demonstrate that the LOD can reach 0.2%–0.5% and confirm the feasibility of detecting multiple mutations. The advantages include the minimal requirements for clinical sample DNA (such as plasma DNA) and the low-cost of clinical genotyping. This study provides a new method with practical value and contributes to the development of clinical molecule testing.

## Declarations

### Author contribution statement

Wang Jianping, Liu Zipeng, Pan Tengfei; Zhang Song: Conceived and designed the experiments; Performed the experiments; Analyzed and interpreted the data; Contributed reagents, materials, analysis tools or data; Wrote the paper.

### Funding statement

Doctor Jianping Wang was supported by Pearl River Nova Program of Guangzhou [No. 201906010038].

### Data availability statement

Data included in article/supp. material/referenced in article.

### Declaration of interest's statement

The authors declare no conflict of interest.

### Additional information

No additional information is available for this paper.

## Appendix A Supplementary data

20220225HELIYON-D-22-02876-SI-0524.doc 20220225HELIYON-D-22-02876-SI-0524.doc
