# Supplementary material for: A multiple detection method for distinguishing gene mutations based on melting curves of extended quenching probes
Source: Heliyon. 2022 Nov 26;8(11):e11856. doi: 10.1016/j.heliyon.2022.e11856 (PMC9712562; doi:10.1016/j.heliyon.2022.e11856)
Supplement: 20220225HELIYON-D-22-02876-SI-0524.doc [file mmc1.doc]

**Supporting information**

**A Multiple Detection Method for Distinguishing Gene Mutations Based on Melting Curves of Extended Quenching Probes**

Wang Jianping^a, 1^, *, Liu Zipeng^b, 1^, Pan Tengfei^a^, Zhang Song^a^

^a^ R&D Department, Guangzhou Biotron Biotechnology Co., Ltd., Guangzhou 510700, Guangdong, China

^b^ Guangzhou Institute of Pediatrics, Guangzhou Women and Children’s Medical Center, Guangzhou Medical University, Guangzhou 510623, Guangdong, China

* Correspondence author.

E-email: [wangjianping198603@126.com](mailto:wangjianping198603@126.com) (Wang Jianping).

^1^ Equal contributors.

**Supplementary information**

**The sequences and function of primers and probes**

The sequences and the functions of primers and probes were listed in the Supplementary Table S1.

Supplementary Table S1. The sequences of primers and probes used in this paper

| Mutation sites | Sequences (5’–3’) | Functions |
| --- | --- | --- |
| G719 | GGAGAAGCTCCCAACCAAGCTCT | Primer (P-F) |
| G719 | GCCAGGGACCTTACCTTATACACCG | Primer (P-R) |
| G719 | CCGAACGCACCGGAGCG | Upstream probe (UP) |
| G719A | FAM-AGAGGGGCAA GCCAGCACTTTGATCT-C_6_ | Downstream probe (DP) |
| G719S | FAM-TCCGTGTCTG TCAGCACTTTGATCTTTTTG-C_6_ | Downstream probe (DP) |
| G719C | FAM-CAGGACACGA ACAGCACTTTGATCTTTTTG-C_6_ | Downstream probe (DP) |
| EGFR-E19 | TGTCATAGGGACTCTGGATCCCAGA | Primer (P-F) |
| EGFR-E19 | GCAGAAACTCACATCGAGGATTTCCTTGT | Primer (P-R) |
| EGFR-E19 | GAAAGTTAAAATTCCCGTCGCTATCAT | Upstream probe (UP) |
| 1E746_A750del(1) | ROX-GATGGGCTTG AAACATCTCCGAAAGCC-C_6_ | Downstream probe (DP) |
| 2E746_A750del(2) | ROX-ACTCCCCGTT AGACATCTCCGAAAGC-C_6_ | Downstream probe (DP) |
| 3L747_P753>S | ROX-TGACCCAAGG AGGAATCGAAAGCCAAC-C_6_ | Downstream probe (DP) |
| 4E746_T751>I | CY5-CTTGGATGGG AAATATCTCCGAAAGCCAA-C_6_ | Downstream probe (DP) |
| 5E746_T751del | CY5-CGTTACTCCC AGTCTCCGAAAGCCA-C_6_ | Downstream probe (DP) |
| 6E746_T751>A | CY5-AAGGTGACCC AGGCATCTCCGAAAG-C_6_ | Downstream probe (DP) |
| 7E746_S752>A | FAM-AGAGGGGCAA AGGCTCCGAAAGCC-C_6_ | Downstream probe (DP) |
| 8E746_ S752>V | FAM-TCCGTGTCTG AGGTTCCGAAAGCCA-C_6_ | Downstream probe (DP) |
| 9E746_ S752>D | FAM-CAGGACACGA AGGATCCGAAAGCCA-C_6_ | Downstream probe (DP) |
| 10L747_A750>P | ROX-GATGGGCTTG AGGAGCCAACATCTCC-C_6_ | Downstream probe (DP) |
| 11L747_T751>Q | ROX-ACTCCCCGTT AGGAAGCAACATCTCC-C_6_ | Downstream probe (DP) |
| 12L747_E749del | ROX-TGACCCAAGG AGGAATCTCCGAAAGC-C_6_ | Downstream probe (DP) |
| 13L747_T751del | CY5-CTTGGATGGG AGGAACCGAAAGCCA-C_6_ | Downstream probe (DP) |
| 14L747_S752del | CY5-CGTTACTCCC AGGAACCAACATCTCC-C_6_ | Downstream probe (DP) |
| 15L747_A750>P | CY5-AAGGTGACCC AGGAACAGAAAGCCAAC-C_6_ | Downstream probe (DP) |
| T790M | GGCATCTGCCTCACCTCCAC | Primer (P-F) |
| T790M | CCGGACATAGTCCAGGAGGCA | Primer (P-R) |
| T790M | CCGAAGGGCATGAGCTGCT | Upstream probe (UP) |
| T790M | FAM-AGAGGGGCAA ATGATGAGCTGCACG-C_6_ | Downstream probe (DP) |
| 16L747_P753>Q | FAM-TCCGTGTCTG AGGAATCTCCGAAAGC-C_6_ | Downstream probe (DP) |
| S768I | GTGCCTCTCCCTCCCTCCA | Primer (P-F) |
| S768I | CCAGCAGGCGGCACACG | Primer (P-R) |
| S768I | GAAGCCTACGTGATGGCCAC | Upstream probe (UP) |
| S768I | FAM-CAGGACACGA TCGTGGACAACCCC-C_6_ | Downstream probe (DP) |
| E20ins | TCCCTCCCTCCAGGAAGCC | Primer (P-F) |
| E20ins | GTGAGGCAGATGCCCAGCA | Primer (P-R) |
| E20insH | GCGTGGACAACCCCCAT | Upstream probe (UP) |
| H773_V774insH | ROX-GATGGGCTTG CCACGTGTGCCG--C_6_ | Downstream probe (DP) |
| E20insG | GGCACACGTGGGGGTC | Upstream probe (UP) |
| D770_N771insG | ROX-ACTCCCCGTT TACCGTCCACGCT-C_6_ | Downstream probe (DP) |
| L858R | TGCATGGTATTCTTTCTCTTCCGCACC | Primer (P-F) |
| L858R | GGAACGTACTGGTGAAAACACCGC | Primer (P-R) |
| L858R | CATGTCAAGATCACAGATTTTGGGCC | Upstream probe (UP) |
| L858R | ROX-TGACCCAAGG GGGCCAAACTGCTG-C_6_ | Downstream probe (DP) |
| C797S | ACCGTGCAGCTCATCACGC | Primer (P-F) |
| C797S | AGCCAATATTGTCTTTGTGTTCCCGGA | Primer (P-R) |
| C797S | AGCTCATGCCCTTCGGCC | Upstream probe (UP) |
| C797S | CY5-CTTGGATGGG TGCCTCCTGGACTATG-C_6_ | Downstream probe (DP) |
| E20insASV | CTACGTGATGGCCAGCGTT | Upstream probe (UP) |
| V769_D770insASV | CY5-CGTTACTCCC GGCCAGCGTGGA-C_6_ | Downstream probe (DP) |
| L861Q | GGTGAAAACACCGCAGCATGTCAA | Primer (P-F) |
| L861Q | ACTTTGCCTCCTTCTGCATGGTATTCT | Primer (P-R) |
| L861Q | TTCTCTTCCGCACCCAGCC | Upstream probe (UP) |
| L861Q | CY5-AAGGTGACCC TGTTTGGCCAGCC-C_6_ | Downstream probe (DP) |
| FAM-85.0°C | ACCGCCCCAGCIGtCICICGCGCATGTCGTGTCCTG-BHQ1 | Quenching probe (QP) |
| FAM-74.5°C | ACAICIAIAICIGtCICICGACATGCAGACACGGA-BHQ1 | Quenching probe (QP) |
| FAM-69.5°C | ACAICIAIAICIGtTICICITGTTGCCCCTCT-BHQ1 | Quenching probe (QP) |
| ROX-87.5°C | ACCGCCCCAGCIGtCICICGCGCATGCCTTGGGTCA-BHQ2 | Quenching probe (QP) |
| ROX-78.5°C | ACAICIAIAICIGtCICICGACATGAACGGGGAGT-BHQ2 | Quenching probe (QP) |
| ROX-71.2°C | ACAICIAIAICIGtTICICITGCAAGCCCATC-BHQ2 | Quenching probe (QP) |
| CY5-86.5°C | ACCGCCCCAGCIGtCICICGCGCATGGGGTCACCTT-BHQ2 | Quenching probe (QP) |
| CY5-76.5°C | ACAICIAIAICIGtCICICGACATGGGGAGTAACG-BHQ2 | Quenching probe (QP) |
| CY5-69.5°C | ACAICIAIAICIGtTICICITGCCCATCCAAG-BHQ2 | Quenching probe (QP) |

**The working concentration of primers and probes**

The concentration of primers (P-Fs, P-Rs), upstream probes (UPs), fluorescent probes (DPs) and quenching probes (QPs) were listed in the Supplementary Table S2 when they work in the reaction.

Supplementary Table S2. The working concentration of sequences used in this paper

| Name of sequences | Working concentration | Name of sequences | Working concentration |
| --- | --- | --- | --- |
| G719-F | 200 nmol/L | S768I-UP | 500 nmol/L |
| G719-R | 200 nmol/L | S768I-DP | 600 nmol/L |
| G719-UP | 300 nmol/L | E20ins-F | 200 nmol/L |
| G719A-DP | 400 nmol/L | E20ins-R | 200 nmol/L |
| G719S-DP | 400 nmol/L | E20insH-UP | 100 nmol/L |
| G719C-DP | 400 nmol/L | H773_V774insH-DP | 400 nmol/L |
| EGFR-E19 | 150 nmol/L | E20insG-UP | 200 nmol/L |
| EGFR-E19 | 150 nmol/L | D770_N771insG-DP | 600 nmol/L |
| EGFR-E19-UP | 300 nmol/L | L858R-F | 200 nmol/L |
| 1E746_A750del(1) -DP | 300 nmol/L | L858R-R | 200 nmol/L |
| 2E746_A750del(2) -DP | 300 nmol/L | L858R-UP | 500 nmol/L |
| 3L747_P753>S-DP | 300 nmol/L | L858R-DP | 500 nmol/L |
| 4E746_T751>I-DP | 300 nmol/L | C797S-F | 100 nmol/L |
| 5E746_T751del-DP | 300 nmol/L | C797S-R | 100 nmol/L |
| 6E746_T751>A-DP | 300 nmol/L | C797S-UP | 100 nmol/L |
| 7E746_S752>A-DP | 500 nmol/L | C797S-DP | 500 nmol/L |
| 8E746_ S752>V-DP | 300 nmol/L | E20insASV-UP | 500 nmol/L |
| 9E746_ S752>D-DP | 100 nmol/L | V769_D770insASV-DP | 600 nmol/L |
| 10L747_A750>P-DP | 300 nmol/L | L861Q-F | 200 nmol/L |
| 11L747_T751>Q-DP | 300 nmol/L | L861Q-R | 200 nmol/L |
| 12L747_E749del-DP | 300 nmol/L | L861Q-UP | 500 nmol/L |
| 13L747_T751del-DP | 300 nmol/L | L861Q-DP | 600 nmol/L |
| 14L747_S752del-DP | 300 nmol/L | QPs-FAM-85.0°C | 500 nmol/L |
| 15L747_A750>P-DP | 300 nmol/L | QPs-FAM-74.5°C | 500 nmol/L |
| T790M-F | 50 nmol/L | QPs-FAM-69.5°C | 500 nmol/L |
| T790M-R | 50 nmol/L | QPs-ROX-87.5°C | 500 nmol/L |
| T790M-UP | 200 nmol/L | QPs-ROX-78.5°C | 500 nmol/L |
| T790M-DP | 300 nmol/L | QPs-ROX-71.2°C | 500 nmol/L |
| 16L747_P753>Q-DP | 300 nmol/L | QPs-CY5-86.5°C | 500 nmol/L |
| S768I-F | 100 nmol/L | QPs-CY5-76.5°C | 500 nmol/L |
| S768I-R | 100 nmol/L | QPs-CY5-69.5°C | 500 nmol/L |

**Detection of the samples using the digital PCR method**

Prepare 25 μL mixture for digital PCR system, which included: 1× PCR buffer, 200 nmol/L primers, 100 nmol/L fluorescent probes, 2.0 U Taq DNA polymerase, and 2 μL template from the clinical samples. The reaction procedure was as follows: 2 minutes at 95 °C, 10 seconds at 95 °C, 40 seconds at 58 °C, 20 seconds at 72 °C, 45 cycles. The results of clinical samples were analyzed by Naica^TM^ Crystal digital PCR system.

**The precision of this method**

To verify the precision of our method, samples with concentrations of 0.2% (L858R), 0.5% (D770_N771insG) and 0% were chosen for each of two mutation sites, and eight repeated tests were completed for each of the samples. The results were shown in Supplementary Figure S1.


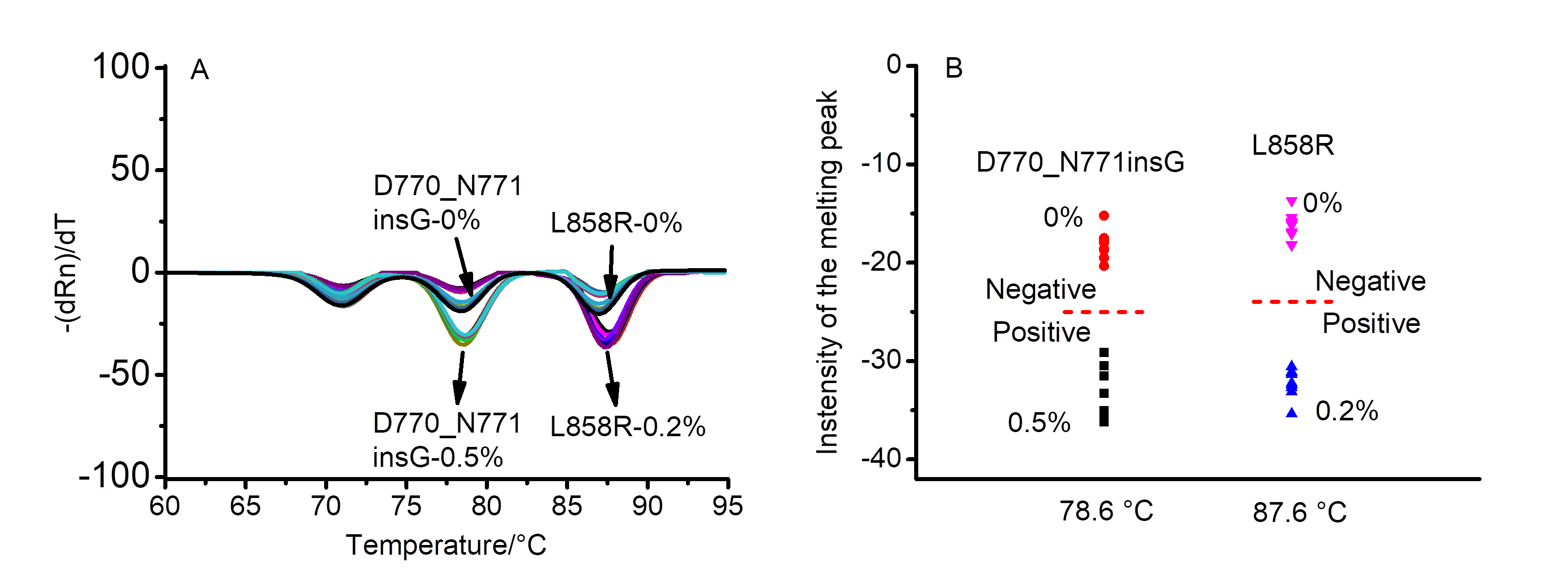


Supplementary Figure S1. Result of 8 repeated tests in this method. S1A represents the result of the positive samples (0.5% D770_N771insG and 0.2% L858R). S1B represents the different intensity of the melting peaks between the positive (0.5% D770_N771insG and 0.2% L858R) and the negative (wild sample).

**The clinical information of samples**

The detailed information of all clinical samples was listed in the Supplementary Table S3.

Supplementary Table S3. The clinical information of 9 samples (Lung cancer).

| Number | Sample ID | Cancer Typing | The detecting result | |
| --- | --- | --- | --- | --- |
|  |  |  | Our method | ARMS-PCR |
| 1 | 10782 | Moderately differentiated Adenocarcinomatous | L861Q | L861Q |
| 2 | 10431 | Adenocarcinomatous | L858R | L858R |
| 3 | 10441 | Adenocarcinomatous | L747_P753>S | 19del |
| 4 | 10462 | Adenocarcinomatous | L747_P753>S | 19del |
| 5 | 10471 | Adenocarcinomatous | E746_A750del(2) | 19del |
| 6 | 10403 | Moderately differentiated Adenocarcinomatous | L858R | L858R |
| 7 | 10411 | Well-differentiated Adenocarcinomatous | L858R | L858R |
| 8 | 10413 | Adenocarcinomatous | E746_A750del(2) | 19del |
| 9 | 10414 | Adenocarcinomatous | L861Q | L861Q |

**The ARMS-PCR using the clinical samples**

The mutated DNA of nine clinical samples were detected by ARMS-PCR (AmoyDx EGFR Mutation Test Kit), and the results were shown in Supplementary Figure S2.


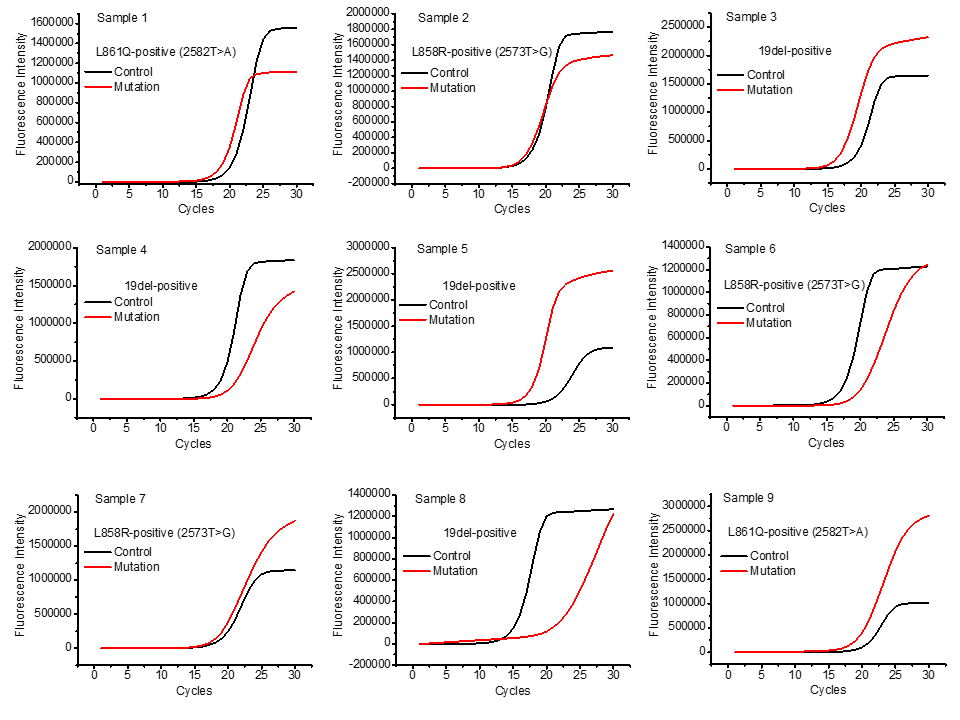


Supplementary Figure S2. Results of nine clinical samples detected using ARMS-PCR method. Of the nine clinical samples, the sample 1 and 9 were L861Q-positive (2582T>A), the sample 2, 6 and 7 were L858R-positive (2573T>G), and the sample 3, 4, 5 and 8 were 19del-positive.
